# Bamboo–Polylactic Acid (PLA) Composite Material for Structural Applications

**DOI:** 10.3390/ma10111286

**Published:** 2017-11-09

**Authors:** Angel Pozo Morales, Alfredo Güemes, Antonio Fernandez-Lopez, Veronica Carcelen Valero, Sonia De La Rosa Llano

**Affiliations:** 1 Department of Aerospace Materials and Manufacturing, Polytechnic University of Madrid, 28031 Madrid, Spain; alfredo.guemes@upm.es (A.G.); antonio.fernandez.lopez@upm.es (A.F.-L.); 2 Institution Abengoa, 41014 Seville, Spain; veronica.carcelen@gmail.com (V.C.V); sonia.delarosa@abengoa.com (S.D.L.R.L.)

**Keywords:** bamboo fiber, PLA resin, green structural composites, mechanical extraction, RFI process, environmental sustainability

## Abstract

Developing an eco-friendly industry based on green materials, sustainable technologies, and optimum processes with low environmental impact is a general societal goal, but this remains a considerable challenge to achieve. Despite the large number of research on green structural composites, limited investigation into the most appropriate manufacturing methodology to develop a structural material at industrial level has taken place. Laboratory panels have been manufactured with different natural fibers but the methodologies and values obtained could not be extrapolated at industrial level. Bamboo industry panels have increased in the secondary structural sector such as building application, flooring and sport device, because it is one of the cheapest raw materials. At industrial level, the panels are manufactured with only the inner and intermediate region of the bamboo culm. However, it has been found that the mechanical properties of the external shells of bamboo culm are much better than the average cross-sectional properties. Thin strips of bamboo (1.5 mm thick and 1500 mm long) were machined and arranged with the desired lay-up and shape to obtain laminates with specific properties better than those of conventional E-Glass/Epoxy laminates in terms of both strength and stiffness. The strips of bamboo were bonded together by a natural thermoplastic polylactic acid (PLA) matrix to meet biodegradability requirements. The innovative mechanical extraction process developed in this study can extract natural strip reinforcements with high performance, low cost, and high rate, with no negative environmental impact, as no chemical treatments are used. The process can be performed at the industrial level. Furthermore, in order to validate the structural applications of the composite, the mechanical properties were analyzed under ageing conditions. This material could satisfy the requirements for adequate mechanical properties and life cycle costs at industrial sectors such as energy or automotive.

## 1. Introduction

Recycling of conventional thermoset composites at the end of their life cycle is a difficult issue; for example, for out-of-service wind turbine blades, landfill is still the most economic option, but Waste Management Directives are increasingly stricter and will limit this approach in the near future. Mechanical crushing and pyrolysis are investigated as alternative procedures, but the development of new natural-based materials, with adequate mechanical properties and durability, may be a more effective solution. Natural fibers have attracted attention because they deliver high performance in terms of specific mechanical properties, easy availability, renewability, recyclability, CO_2_ neutrality, non-toxicity upon inhalation, direct extraction from nature without further processing, non-abrasion to machines, low energy consumption, and low cost [1,2,3]. Furthermore, natural fibers processed as composite materials with thermosetting or thermoplastic polymers can serve as environmentally friendly alternatives to standard E-Glass composites, wood-reinforced materials, and aluminum alloys. Table 1 shows the properties of the most common natural fibers used in reinforced materials.

As can be observed, the mechanical properties present a broad range of values. These high deviations originate mainly from the following.

- Flaw distributions and fiber discontinuities hinder the process of measuring and estimating the average cross-sectional areas of fiber bundles or elementary fiber [6].
- Many varieties of species exist for a given natural fiber. For example, over 1250 species of bamboo exist worldwide, and all are referred to by the same name “bamboo” [16].
- The average mechanical properties depend on the scale of the specimens considered for the test. The values of the mechanical properties are higher at the microscale than at the macroscale. Wang et al. measured the tensile strength of 1200 MPa for an elementary microscale fiber of bamboo; when considering a bundle of fibers at the macroscale level, the tensile strength was 800 MPa [6].
- The mechanical properties of the fiber are affected by the species, weather conditions, crop production, harvest stage time [15], and section considered for the extraction of the fiber bundles. Verma et al. reported that the tensile strength was 97.9 MPa in the inner region and 237.93 MPa in the outer region of a Moso bamboo culm [17].
- The different processing methodologies used to extract the fiber can promote random weak points, causing premature failure (mechanical extraction) [13]; for an optimal applied concentration, the methodology may improve the mechanical properties (chemical extraction) [18].
- Standards considered to perform tests on fibers are lacking. In general, mechanical tests are performed based on the morphology of the fiber instead of proper standards, which define the dimension of the specimen, speed of the test, and clamping conditions, among other parameters. The specific standard ASTM C-1557-03 exists for measuring the tensile properties of fibers, but in the literature, not all tests have been performed based on this standard.

All these conditions complicate the proper comparison of natural fibers among different studies, so definitive selection of the optimal fiber type requires not only a bibliographical analysis, but also mechanical tests performed on a wide sample test batch (minimum of 20 fibers) of specific natural fibers in order to define the average and standard deviation of a given property. The number of mechanical tests suggested here is higher than usual because of the irregular morphology of natural reinforcement fibers, which promotes unusually high dispersion in measurements.

Despite the promising results found in the literature for the mechanical properties of natural fibers, the final properties of natural fiber-reinforced composites are generally poorer than expected. Hence, the final products are used only in secondary markets (furniture, flooring, sport devices, and packaging), with limited applications in structural designs [6]. Table 2 and Table 3 summarize the main values of the mechanical properties of natural fiber-reinforced composites published by companies or in recent studies. Notably, most of the reported values have not yet been verified.

Based on the maximum tensile mechanical properties of flax fibers found in the literature (*σ*_11_: Tensile strength; *E*_11_: Young’s modulus in fiber direction) (*σ*_11_ = 1445 MPa, *E*_11_ = 160 GPa), the tensile properties of the epoxy resin (*σ*_11_ = 60 MPa, *E*_11_ = 3.5 GPa) and a fiber volume fraction V_fiber_ of approximately 50%, the tensile mechanical properties of a unidirectional flax-epoxy composite are determined by the rule of mixtures as approximately 752.5 MPa and 81.8 GPa).

However, the maximum values published by the companies Lineo and Bcom in their products are 383 MPa and 35 GPa and thus much lower than the theoretical values calculated by the rule of mixtures. These reflect that some issues can promote the premature failure of the composite, such as the flaw distribution introduced during the manufacturing process of the isolated fiber, the low interfacial strength, the non-continuity of the natural fibers compared to synthetic ones, such as glass fibers, and the improper measurement of some experimental results found in the literature.

Hence, in order to develop data sheets for each fiber, it is necessary to measure the properties according to standards. These standards should be developed based on all the parameters mentioned above to reduce their influence on the final measurements.

Other issues to overcome are the moisture sensitivity [34], poor fire resistance [35], restricted operational temperature from the low glass transition temperature (*T*_g_ = 65 °C) of biopolymers [36], restricted maximum processing temperature from the maximum degradation temperature of natural fibers (~200 °C) [2], durability, and degradation under ultraviolet light [15,37,38].

In terms of cost, products currently on the market cost more than expected based on the price of the raw material at 16 €/m^2^, because of the large initial investments paid by the developing companies [23].

It should be noted that composites based on non-biodegradable polymers have better mechanical properties for resin contents of approximately 50%. However, such materials cannot be considered eco-friendly alternatives, mainly because of the non-biodegradable behavior of the reinforcing polymers. Other alternatives, such as bio-based epoxy resin could be considered to meet the requirements of recyclability, but different end of life scenarios should be analyzed considering this resin [39].

The main scientific interest has been in the development of a biodegradable composite material that can decrease carbon footprints and petroleum dependence; however, this remains a challenge. Indeed, the biodegradable products found on the market have high costs and poor properties (*σ*_11_ = 110 MPa, *E*_11_ = 14 GPa).

Therefore, the main aim of this study is the manufacture of a fully biodegradable composite material for structural applications. For that purpose, bamboo has been selected to manufacture a composite material based on strips of bamboo from the external thickness of the culm and PLA film matrix, because bamboo is one of the most promising candidates based on engineering criteria related to the mechanical properties, morphology conditions and cost. To extract strips from the external thickness of the bamboo culm with high dimensional tolerance, a novelty extraction methodology was completely performed because at industrial level the methodology used provides bamboo-laminated panels with mechanical properties lower than the expected based on the mechanical properties of the natural fiber. This is mainly because the section considered is the inner and intermediate region and the sanding and cutting processes promote strips with discontinuities along the length and thickness.

PLA resin fulfills the biodegradable requirements and it is compatible with the manufacturing process RFI (resin film infusion) and is considered to obtain high mechanical properties. The manufacturing process was optimized based on the parameters involved. Furthermore, to estimate the permissibility of the material, the mechanical properties were analyzed under room temperature and ageing conditions.

## 2. Materials

In terms of origin, natural fibers based on plants can be grouped into woods, stalks, fruits, grasses and reeds, bast, leaves, and seeds. The most commonly used materials in composite applications include bast (flax, hemp, jute, and kenaf), leaf fibers (sisal), and grass (bamboo) [40].

### 2.1. Bamboo

Bamboo, a hollow culm, is a perennial evergreen belonging to the grass family. It has two different areas called nodes and internodes along the length. The distance between each node varies depending on the bamboo species [41]. Among natural fiber plants, bamboo has attracted considerable attention in recent years as a sustainable structural material for different applications (building construction, housing, flooring, alternative fillers, automotive, and furniture) [42] because it has low density, good mechanical properties, and low cost.

As can be seen in Table 4, the designs and solutions proposed at the industrial level (flake board, bamboo flooring, laminated bamboo lumber, and laminated bamboo veneer) have poor mechanical properties. This is mainly because the thickness considered for the manufacture of the lumber or veneer is the full thickness of the culm wall, rather than using only the external thickness, which shows better mechanical properties.

As noted before, one issue related to natural fibers is the number of species per genus, as different species provide different properties. Hence, in this study, only the most common species used in building applications, Moso and Guadua, are considered.

#### Morphological Study

**Figure 1:**
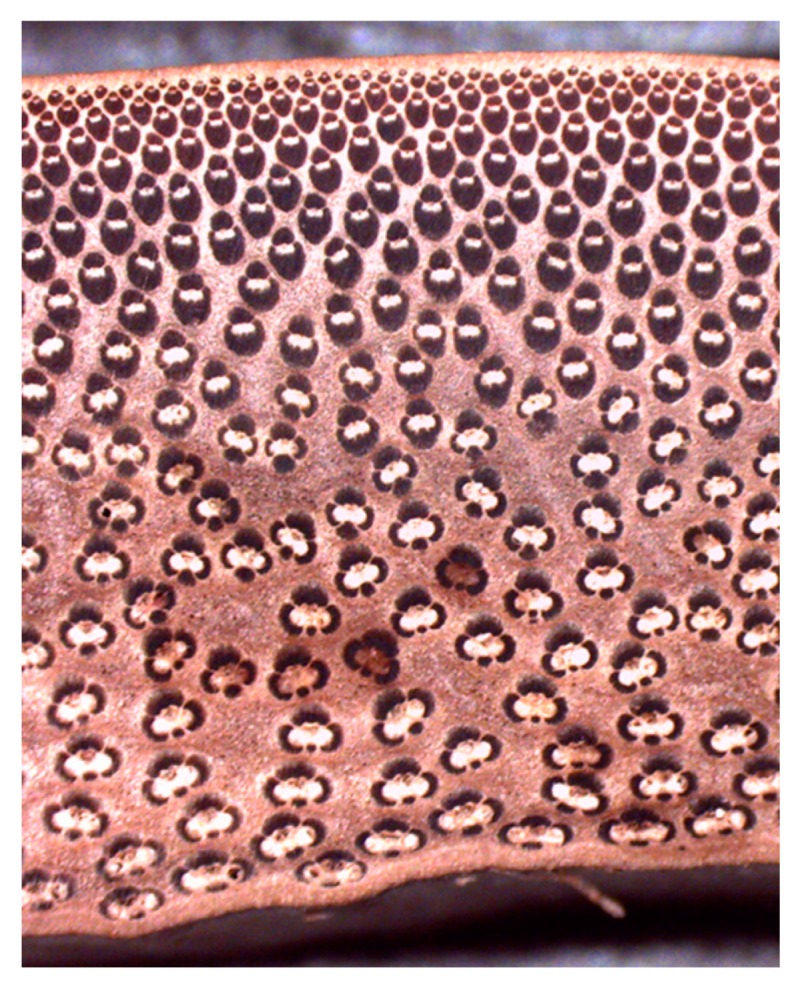
Bamboo cross section.

The main morphological characteristic is that the density of fiber bundles varies from the outer to inner boundary of a cross-section of bamboo. At the outer boundary, the fibers are compact and more numerous than those at the inner boundary. Moreover, depending upon the position and not only the fiber content, their sizes and shapes also vary, as shown in Figure 1.

Table 5 shows the morphological characteristics for both bamboo species.

Greater internodal lengths correspond to lower node influences on the final mechanical properties of the composite, because fewer numbers of internode sections will be included in the final composite panels. Likewise, larger culm diameters correspond to higher numbers of extracted strips. Moreover, during the extraction process, only the outer section of 3 mm in thickness is considered, as it provides the best structural properties. Preliminary results performed in this study show that the inner region has up to 40% lower mechanical properties compared with the average value from the outer region due to increase in the volume fraction of bamboo fibers. These values are in line with those in other studies on the bamboo culm [17,54,55]. Furthermore, the section of the bamboo culm considered to perform the extraction process was the middle region called “Baza”, due to it having a good balance between in mechanical properties and morphology conditions (thickness wall, culm diameter and length).

### 2.2. Fiber Strip Properties

**Figure 2:**
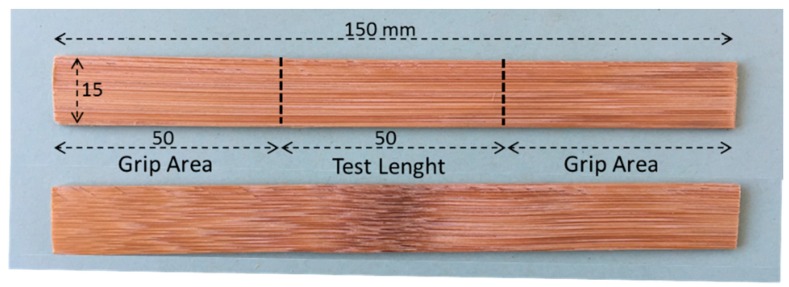
Tensile test specimen.

Four-year-old green bamboo (Guadua Angustifolia Kunth) and Moso (Phyllostachys edulis) culms were obtained from Colombia and China at the company Bambusa [53]. The two species and specimens with and without node influence were investigated to obtain the Young’s moduli (*E*_11_) and tensile strengths (*σ*_11_) based on the standard UNE-EN ISO 527-5 [56] to determine the better species for structural applications. Five specimens Figure 2 were tested for each species and type. The tests were performed using a testing machine (810, MTS, Madrid, Spain) equipped with a 100-kN load cell, 25-mm extensometer, and hydraulic grips.

The specimen size was 150 mm × 15 mm × 1.5 mm, shorter than that for standard specimens, to obtain properties without any node influence. The specimens with nodes are considered because they quantify the changes in mechanical properties from wrinkles and fiber discontinuities at the nodes.

**Figure 3:**
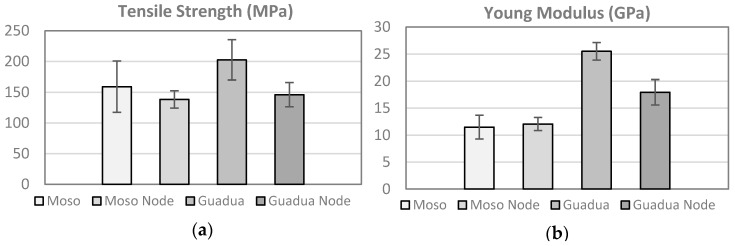
Tensile properties of bamboo strips, (**a**) Tensile Strength and (**b**) Young Modulus.

The Guadua bamboo has better mechanical properties, with an average tensile strength of 202.7 MPa (coefficient of variation COV = 16.2%) and an average Young’s modulus of 25.52 GPa (COV = 6.4%) Figure 3. The mechanical properties are approximately 30% lower in the specimens with nodes. 

As conclusion of the morphology and test strip properties, the Guadua species has been selected as the better candidate to manufacture the bamboo composite.

### 2.3. Innovative Extraction Process

The extraction process of the fiber from the raw material is critical to obtain good mechanical properties in a natural fiber-reinforced composite. Currently, chemical and mechanical processes are used in the industry, but the extraction of good-quality fiber remains a challenge. In this study, an innovative mechanical extraction process is developed to extract strips from the outer section of the culm.

#### 2.3.1. Dimensions of the Strips Extracted

In order to ensure good mechanical properties and to ensure sufficient drapability to manufacture curved panels with a radius of curvature of <500 mm), it is necessary to define the optimum dimensions of the strips. For this purpose, tensile tests based on UNE-EN ISO 527-5 [56] using strips with node influence; widths of 25, 15, and 10 mm; and thicknesses of 2.5, 2, 1.5, and 1 mm were performed.

**Figure 4:**
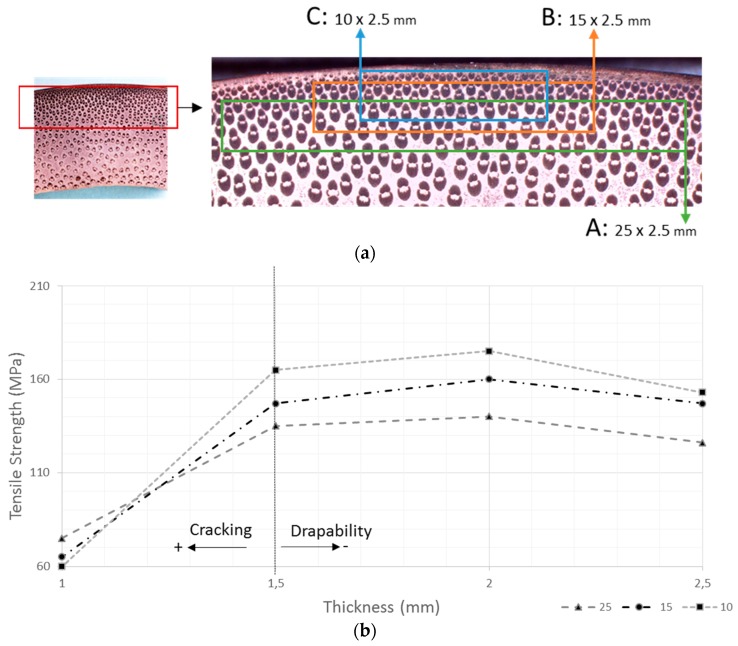
(**a**) Extraction zones of the strips; (**b**) Relationship between the mechanical properties and dimensions of the strips.

As the fiber density is higher in the outer section, the width of the strip and the mechanical properties are related. As can be seen in Figure 4, the strips with widths of 10 mm have better properties (Zone C), because strips from only the external section are extracted. However, it should be considered that the strips are laminated close together; therefore, when using strips of 10 mm in thickness, the number of strip joints used to manufacture a layer of bamboo strips is higher. Because the joints are points of weakness in the layer, the final mechanical properties in the composites are lower. The strips with widths of 25 mm (Zone A) have poorer properties because the strips are extracted from the medium section with lower volumes of fiber.

The ideal width of the strip is approximately 15 mm (Zone B), where the external section comprises the majority of the extracted strips.

The drapability is related to the thickness, and thus the stiffness, of the bamboo strips. In order to ensure good drapability and avoid decreasing the mechanical properties, the thickness range should be 1.5–2 mm. A thickness lower than 1.5 mm decreases the mechanical properties because microcracks are formed along the strip during the extraction process.

The length is considered based on the criterion of easy handling. Based on this, the bamboo strip dimensions selected to manufacture the panels of the bamboo composite are the length of 1500 mm, width of 15 mm, and thickness of 1.5 mm. The final mechanical properties of the composite are influenced by the node, instead of only the internodal area considered in other research [17].

#### 2.3.2. Mechanical Extraction Process

In order to ensure the dimensional accuracy of the bamboo strips, a cutting process was performed instead of the planning and grinding processes commonly used in the wood industry to manufacture veneers or panels.

**Figure 5:**
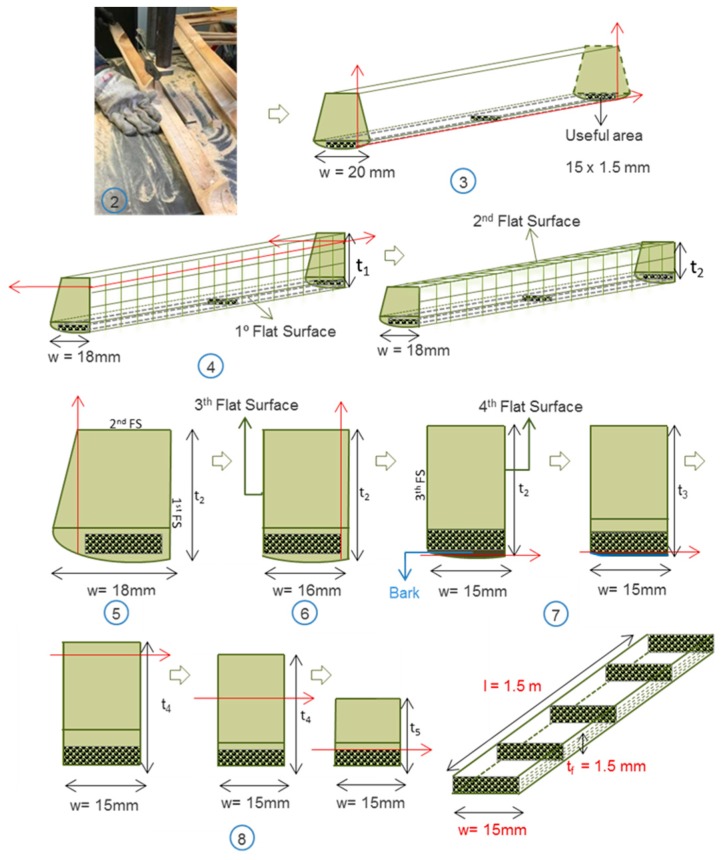

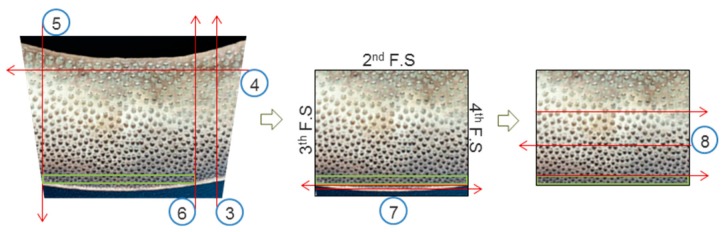
Mechanical extraction process.

The mechanical extraction process consists of the following steps Figure 5:

1. Using a saw band, the bamboo culm is cut every 1.5 m.
2. Using a saw band, the culm is cut longitudinally in slides (1.5 m × 20 mm × wall thickness). The width deviates by up to 2 mm over the length.
3. The slide is cut along the length of the external area to obtain the first flat surface (1st). This cut is made by a saw band and requires later machining by a saw disk.
4. The inner area is cut using the first flat surface as a reference to obtain the second flat surface (2nd) using a saw disk.
5. The width of the slide is cut in order to obtain the third flat surface (3rd) using the previous flat surfaces (1st and 2nd) as references.
6. The width of the slide is cut again to obtain the fourth flat surface (4th) using the previous 2nd and 3rd flat surfaces as references. Then, the final width of the strip is obtained (1.5 m × 15 mm × wall thickness).
7. After obtaining the width with three flat surfaces, the next step is the machining of the slide to obtain the useful area. For this purpose, the bark is removed in two cutting process along the length, to avoid removing excessive external area. Quality control is performed by visual inspection.
8. Finally, the inner area is reduced to obtain the final strip (1.5 m × 15 mm × 1.5 mm). In order to avoid microcracks in the strip, this step should be done in two steps.

**Figure 6:**
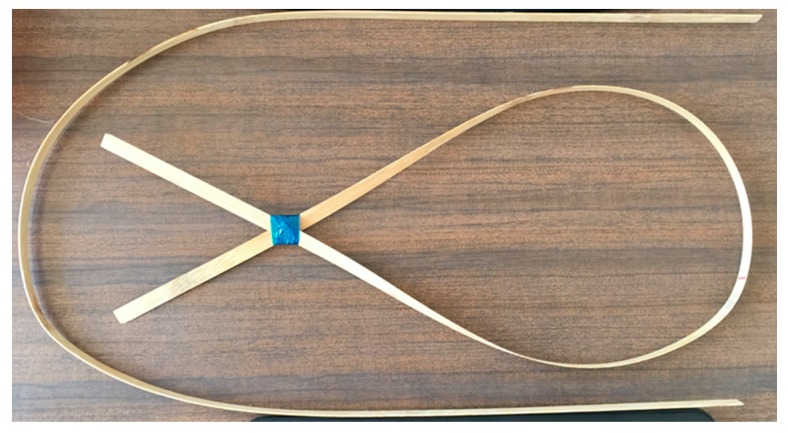
Extracted strip of Guadua bamboo.

The mechanical extraction process provides strips with high dimensional accuracy and fewer microcracks along the thickness compared to strips obtained by planning and grinding as is generally used in industry Figure 6.

Furthermore, before performing the mechanical extraction, quality control was established based on the age (4 years), culm diameter (110–120 mm), internodal distance (>300 mm), wall thickness (>20 mm), and middle longitudinal section (“Baza”) of the raw material, to minimize the influence of variations on the properties and decrease the standard deviations in dimensions [53]. Finally, optimal storage conditions were determined to avoid rotten batches and moisture content of bamboo strips extracted were reduced by oven at 50 °C for 24 h.

### 2.4. Matrix

Regarding the polymer, the main selection requirements were full biodegradability, bio-based sourcing, commercial availability, and compatibility with the manufacturing process. Based on these, polylactic acid (PLA) was considered the most suitable candidate as a fully biodegradable thermoplastic polymer. PLA belongs to the family of aliphatic polyesters with the basic constitutional unit of lactic acid. It can be obtained via the bacterial fermentation of corn [57]. Ingeo PLA Biopolymer 4032D PLA as 0.025-mm-thick film sheets were considered [36] for use in the selected manufacturing process of resin film infusion. Differential scanning calorimetry (DSC) analysis was performed to obtain the melting properties of the PLA (Tm: melting temperature and tm: melting time). The main limitation of PLA is its low *T*_g_ of 60–65 °C, which defines its future applicability.

## 3. Biocomposite Manufacturing Process

Resin film infusion was utilized to manufacture the composite panels. To obtain high performance, this process was optimized based on the characteristics of the materials.

### 3.1. Manual Lay-Up

**Figure 7:**
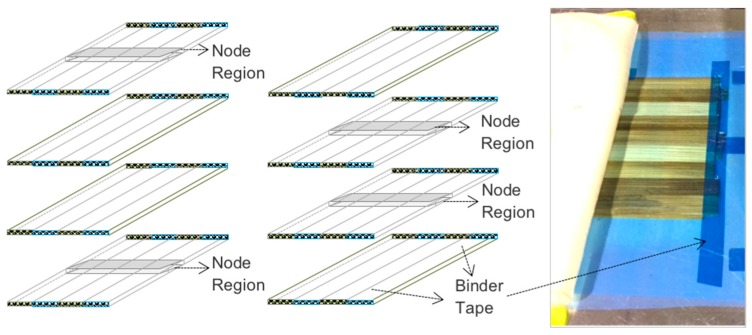
Optimum distribution of the nodes.

A manual lay-up process was performed to manufacture layers of bamboo strips while avoiding gaps and overlaps, positioned and maintained the strips during the lay-up process, an epoxy binder and tape adhesive for high temperatures were applied at the edges of the strips, so that edges would be removed during the machining of the panel. Moreover, the optimum distribution of the nodes was considered to reduce their influence on the composite properties. The nodes were distributed in the middle area of the outer or inner layers and interleaved with node-free layers, such that the layers without nodes stabilized those with nodes and hence reduced the number of weak points in the panel Figure 7.

**Figure 8:**
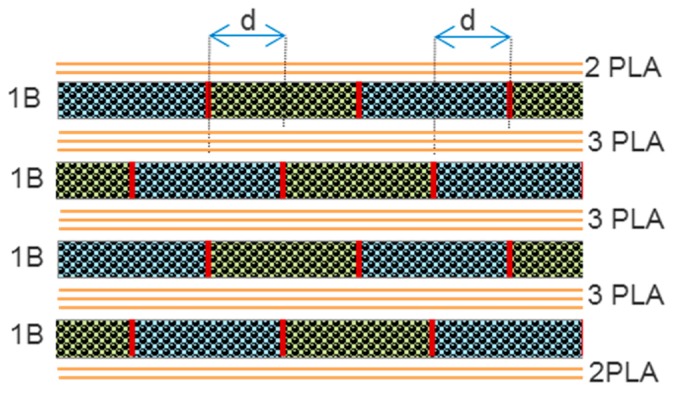
Lay-up process.

Moreover, to avoid weak points along the thickness, each layer was distributed with an edge offset of 7.5 mm (d) between the bottom and top layer Figure 8.

Finally, to minimize the density and increase the fiber content of the composite, only three layers of PLA resin were used for each interface.

### 3.2. Melting Process

**Figure 9:**
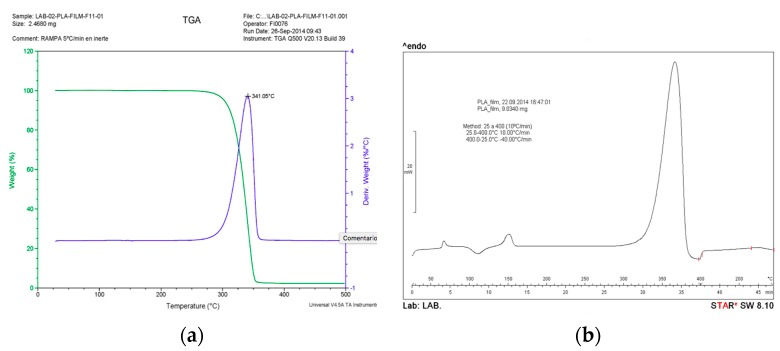
(**a**) Thermogravimetric analysis (TGA) and (**b**) Differential scanning calorimetry (DSC) of polylactic acid (PLA) polymer.

In order to reduce the porosity, avoid un-melted areas, and degrade the bamboo, a thermal cycling study was performed. Thermogravimetric analysis (TGA) technique was used to analyze the thermal stability of the PLA film Figure 9. The measurements were carried out using Thermal Analyzer (Q500 V20.13, TA Instruments, Madrid, Spain) equipment in nitrogen atmosphere. It was conducted in a programmed temperature range from 20 to 500 °C at a heating rate of 5 °C/min and sample weights were approximately 2.50 mg. The results show that the degradation takes place at around 341 °C. Differential scanning calorimetry (DSC) technique was used to determine the melting parameters and *T*_g_ of the PLA film. The measurements were carried out using (DSC 1 Mettler Toledo Star SW 8.10, Madrid, Spain) equipment. It was conducted in a programmed temperature range from 20 to 400 °C at a heating rate of 10 °C/min and sample weights were approximately 9.0 mg. The results show that the melting temperature and time is around (165 °C and 8 min) and the *T*_g_ around 60–65 °C.

Based on the literature [48,58], TGA curves of bamboo fiber, show that there is an initial peak around 40–100 °C, where there is a loss in weight around 3.8% attributed to the vaporization of the water and a second peak around 200–220 °C, where the degradation of the fiber takes place. 

**Figure 10:**
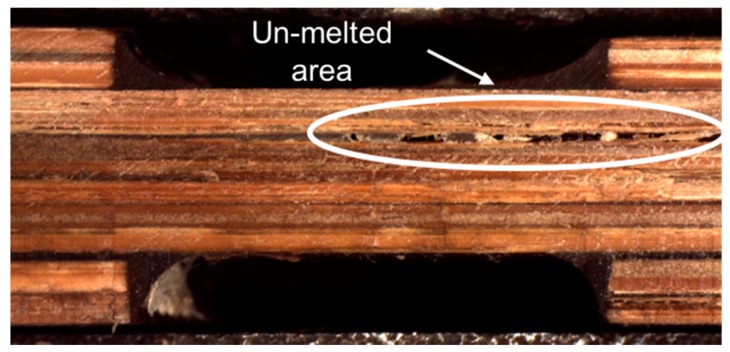
Un-melted area because of the low temperature and time during the melting cycle.

As results:

- The bamboo strips are degraded for increases in temperature or time beyond 195 °C or 20 min at that temperature, respectively. This conclusion was based on mechanical result obtained with panels manufactured at those conditions. The decreasing of the tensile properties was around 35% compared with the mean values obtained at optimum conditions. Based on the result, a TGA should be developed to determine the temperature of degradation of the bamboo–PLA composite.
- Un-melted areas exist for temperatures or times lower than those of the optimum conditions. This conclusion was based on visual inspections of the interphases of the panels manufactured under these conditions Figure 10.

**Figure 11:**
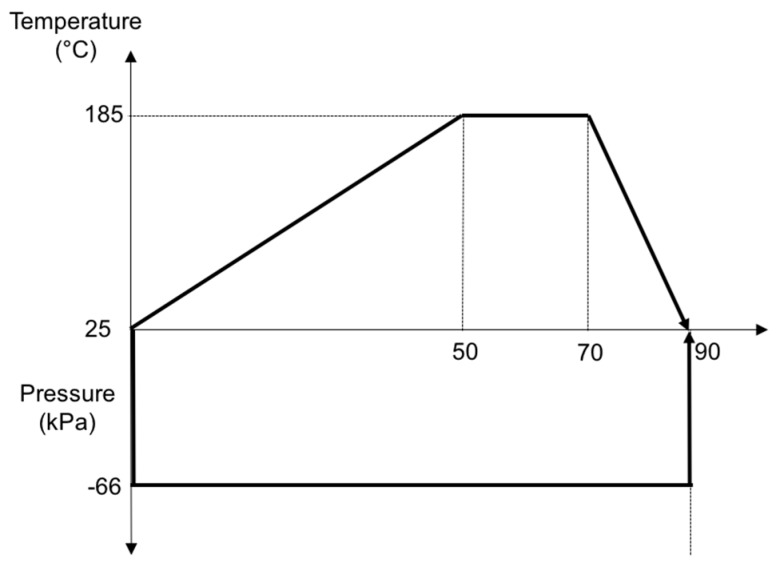
Melting cycle of bamboo–PLA composite.

Therefore, the range of the temperature and time should be between 180–185 °C for ~20 min. These values are in accord with those in other studies on the PLA melting process [27]. The ramp of heating 3.2 °C/min was considered to avoid thermal shock and ensure a uniform heating phase, but a deep analysis should be done to ensure that the bamboo strips are not degraded with this long heating phase, considering other options such as putting into the oven the panels when it reaches 185 °C for 20 min. Moreover, it should be remarked that the melting time of the cycle was related to the thickness of the panel, so for manufacturing complex and large demonstrators the optimum parameters should be re-analyzed. Figure 11

**Figure 12:**
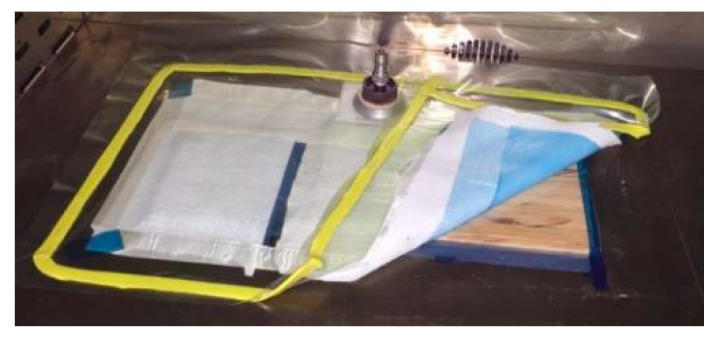
Resin film infusion vacuum assistance for manufacturing bamboo–PLA composite.

Resin Film Infusion (RFI) was selected as manufacturing standard process, so a vacuum bag was considered to apply the pressure and extract pores and volatiles Figure 12. Higher pressure should improve the fiber and matrix integration, as it decreases the porosity. However, as bamboo–PLA is applied as a low-cost solution, only out-of-autoclave manufacturing methods are considered.

### 3.3. Panel Machining

**Figure 13:**
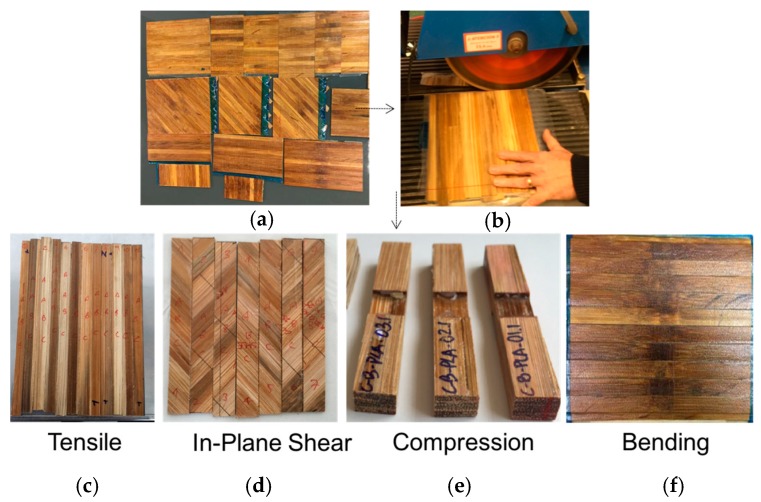
Specimens manufactured from bamboo–PLA composite, (**a**) Manufactured panels; (**b**) Panel machining; (**c**) Tensile specimens; (**d**) In Plane Shear specimens; (**e**) Compression specimens; (**f**) Bending specimens.

Finally, the manufactured panels were cut with a saw disk, the dimensions of each specimen test were based on standards Figure 13.

### 3.4. Demonstrations

**Figure 14:**
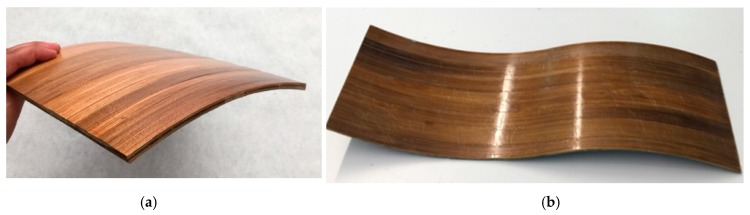
Demonstration panels manufactured from bamboo–PLA composite, (**a**) Demonstrator 1 cylinder concave shape; (**b**) Demonstrator 2 concave and convex shape.

Two demonstration laminate pieces Figure 14 with curved geometries were manufactured to prove the possibility of producing structures with complex geometries, which is an important issue for future applications.

**Figure 15:**
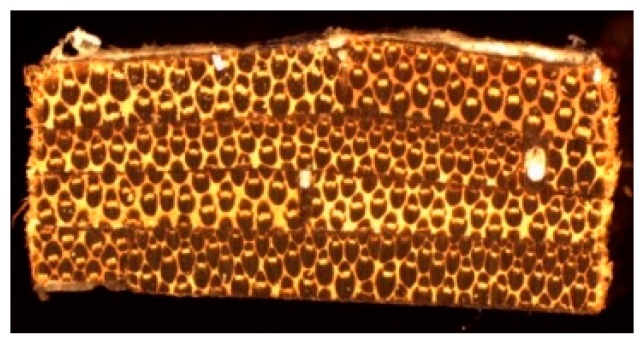
Transverse cross-section of bamboo–PLA composite.

Figure 15 shows the transversal section of the bamboo–PLA composite. In order to determine the fiber content, an inspection using a micrographic analysis on Image-J software was used.

The amount of PLA is low, because only the necessary film layers were used to bond the strips. In fact, the resin of these composites is the lignin where the bamboo bundles are embedded.

- Fiber content of full composite material: 63%
- Lignin content of full composite material: 33.5%
- Resin content (PLA): 3.5 %

## 4. Experimental Results

For a comprehensive study, 20 standard specimens were manufactured for each mechanical test (tensile, compression, in-plane shear, and four-point bending). The tests were conducted according to respective standards using an testing machine (810, MTS, Madrid, Spain) equipped with 10-kN and 100-kN load cells, a 25-mm extensometer, and hydraulic grips and all of the quasi-static tests were conducted at room temperature of 25 °C and average humidity of 50%, under atmospheric pressure.

Furthermore, to define the operation restrictions, the mechanical properties were analyzed under environmental conditions. The purpose of these tests is to demonstrate the ability of the material to withstand the humidity and temperature. Ageing conditions (steady temperature of 50 °C and relative humidity of 95% for 7 days) were selected based on the diurnal cycle of the storage conditions in a B1 category (Kourou weather), due to requirements of the project (AO/1-7460) of the European Space Agency. The ageing was performed in a climatic chamber (HC 7005, Heraeus Votsch, Madrid, Spain).

Next, a summary of the results obtained during comprehensive tests is presented, comparing the specific mechanical properties obtained for samples submitted to the specified environmental conditions to those from an E-Glass/Epoxy composite material considered as a reference. The mechanical properties of the E-Glass/Epoxy were obtained from a database (ESAComp V4.6, Componeering, Helsinki, Finland), based on ASTM D 3039 [59], at room temperature and relative humidity and non-aged conditions.

The specific properties were calculated using the calculated density of 0.91 g/cm^3^ for the bamboo–PLA composite. The density of composite was measured in accordance with the ASTM D3800 [60] with isopropyl alcohol as an immersed fluid and an electronic balance (A210P, Sartorius, Madrid, Spain). Weights were measured to the nearest 0.0001 g. The average composite density was obtained based on the measurement of 20 specimens.

### 4.1. Tensile Test

Tensile tests were conducted according to the UNE-EN ISO 527-5 [56] standard. The lamination sequences of the specimens were [0°]_4_ and the dimensions were 250 mm × 15 mm × 6 mm.

**Figure 16:**
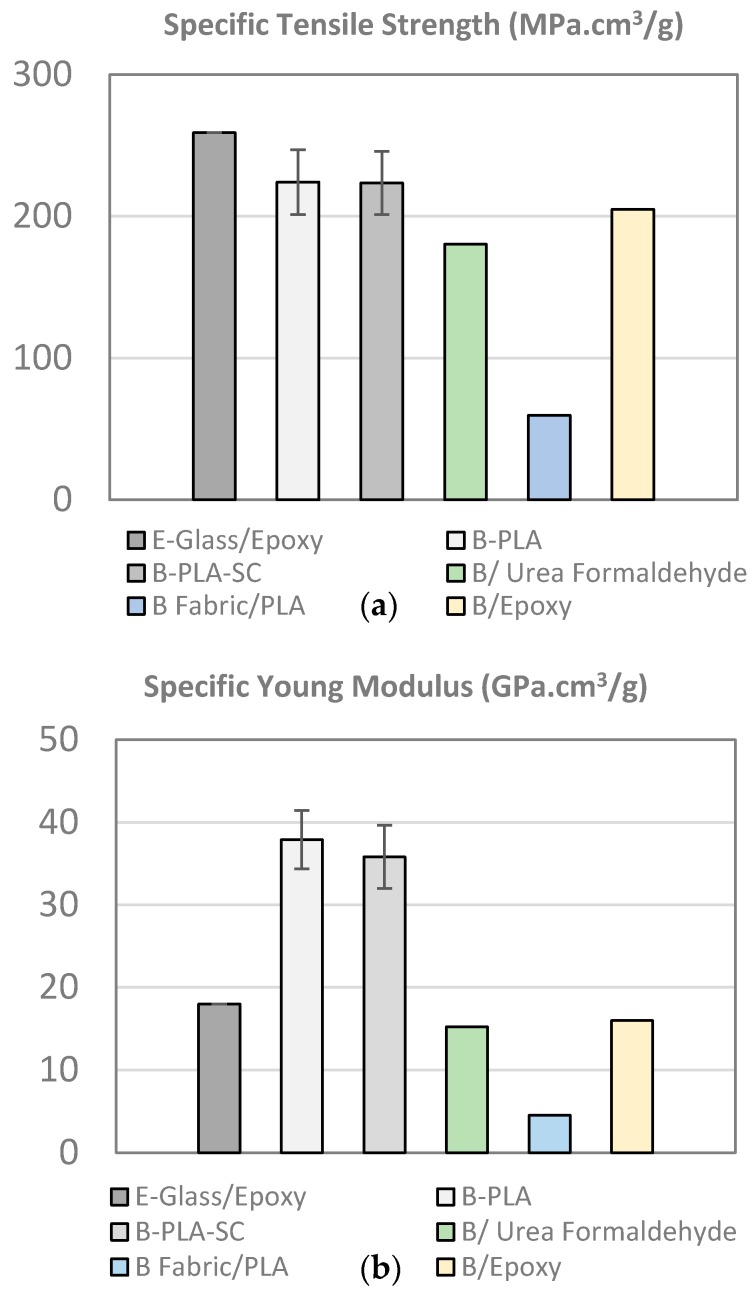
(**a**) Specific tensile strength; (**b**) specific Young modulus (B–PLA: bamboo–PLA non-aged; B–PLA–SC: bamboo–PLA aged; B Fabric/PLA [47]; B/Urea Formaldehyde [43]; B/Epoxy [17]).

Figure 16 show a comparative study between the bamboo–PLA composite manufactured in the present study and the main values founded in the literature for bamboo composites (Table 4). As can be observed, the bamboo–PLA competes with E-Glass/Epoxy in terms of the specific strength and only the Bamboo/epoxy composite manufactured by Verma et al. [17] have a comparable value with the present study. However, the bamboo–PLA composite clearly outperforms the E-Glass/Epoxy composite and the other similar composite in terms of specific stiffness (over 200% with E-Glass/Epoxy, B/Epoxy). 

It should be pointed out that in the study developed by Verma et al. [17] and Hebel et al. [50], where comparable mechanical properties were measured, the section considered for the manufacturing of the laboratory-scale panels was only the internodal area of the culm. Therefore, the influence of the node was not considered. Hence, the mechanical properties of the composite reported by Verma et al. and Hebel et al. cannot be considered as valid to compare with these studies. In the present study, the panels manufactured had 1.5 m of length laying up node and internode region, so the properties obtained can be extrapolated to real size structures. 

The results show that the innovative extraction methodology and the optimization performed in the manufacturing process have a dramatic impact in the specific mechanical properties.

### 4.2. Compression Test

Compression tests were conducted according to the ASTM-D695 standard [61]. The lamination sequences of the specimens were [0°]_4_ and the dimensions were 80 mm × 12.5 mm × 6 mm.

**Figure 17:**
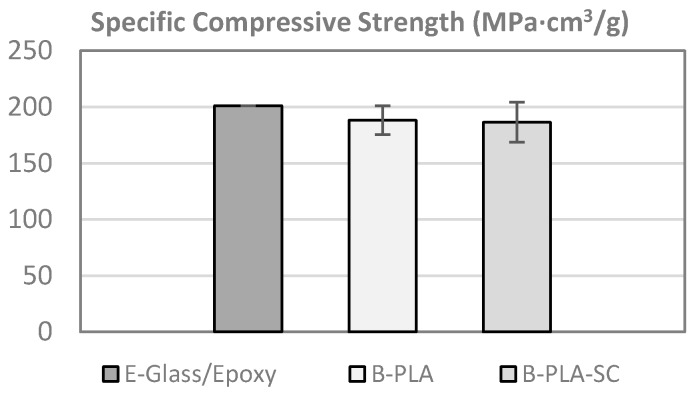
Specific compressive strength (B–PLA: bamboo–PLA non-aged; B–PLA–SC: bamboo–PLA aged).

The specific compressive strength of the bamboo–PLA composite is approximately 84% of the specific tensile strength, similar to the ratio found in aerospace composites. The standard deviation is also aligned with that of the traditional composites at less than 10%. As above, it is comparable to the strength of the reference E-Glass/Epoxy Figure 17.

### 4.3. In-Plane Shear Test

The tests were conducted according to the UNE-EN ISO 14129 standard [62]. The lamination sequences of the specimens were [+45°, −45°]_s_ and the dimensions were 250 mm × 25 mm × 6 mm.

**Figure 18:**
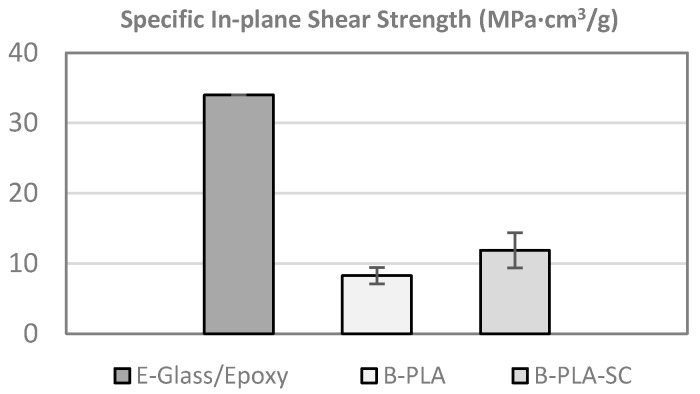
Specific in-plane shear strength (B–PLA: bamboo–PLA non-aged; B–PLA–SC: bamboo–PLA aged).

The specific in-plane shear strength of the bamboo–PLA composite is not comparable to that of the reference Figure 18. This can be attributed to the low adherence between the fiber and matrix, resin-rich areas, and gaps between strips that promote premature failure.

The low adhesion between the bamboo strips and the PLA matrix could be improved by applying chemical or mechanical treatments to the bamboo after extraction. This will be researched in the future. However, it should be noted that it may be impossible to obtain values comparable to the reference, so other resins can be also considered.

### 4.4. Four-Point Bending Test

**Figure 19:**
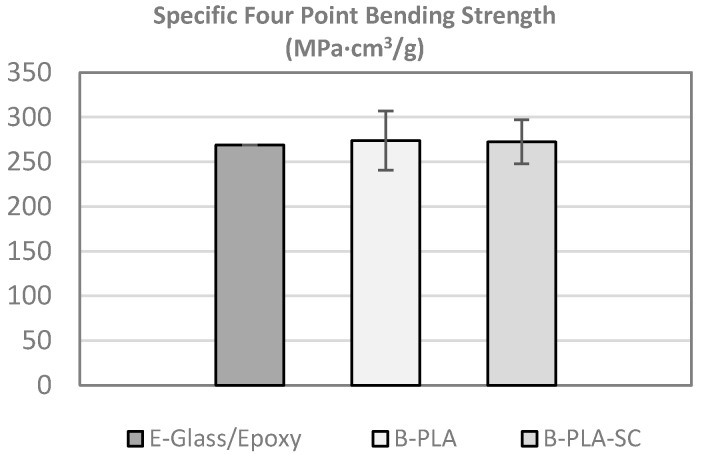
Specific four-point bending strength (B–PLA: bamboo–PLA non-aged; B–PLA–SC: bamboo–PLA aged).

The tests were conducted according to the UNE-EN ISO 14125 standard [63]. The lamination sequences of the specimens were [0°]_3_ and the dimensions were 120 mm × 25 mm × 4.5 mm. The specific flexural strength of the bamboo–PLA composite is similar to that of the reference Figure 19.

It should be noted that the tensile mechanical properties of the strip were 30% less because of the node influence. The optimizations introduced in the manufacturing process compensate for the presence of nodes, while assuring low standard deviations and high averages for specific mechanical properties. These results demonstrate the potential of the material as an eco-friendly alternative.

#### 4.4.1. Ageing Storage Conditions (SC)

After storage in the specified conditions, the specific tensile, compressive, and bending properties are similar to the specific mechanical properties in the non-aged specimens. Therefore, the aging cycle does not influence the mechanical properties.

The specific shear strength was increased by 43% compared to the specific shear strength measured before ageing. In order to understand this value, a deeper study is required, in which it should be considered that the temperature of the storage condition of 50 °C is near the *T*_g_ of the PLA (65 °C), cold crystallization of the PLA upon ageing could be considered as hypothesis of this phenomenon.

To determine the real permissibility of the material, it is be necessary to define the worst ageing conditions, taken into account the moisture absorbed by the composite and its influence on the mechanical properties.

#### 4.4.2. Damage Characterization

To analyze the damage mechanisms in a detailed manner, the fracture surface of the bamboo–PLA composite was observed by scanning electron microscope (S-4800, Hitachi, Tokyo, Japan). Prior to the analysis, the fracture surface of the specimens were coated with a thin layer of gold to get good conductivity The SEM (scanning electron microscope) photomicrograph represents the local morphology of the fracture surface, the different phases (bamboo bundles and lignin resin of the strips and PLA polymer interphase, were detected by hand cropping detection. The analysis of the sequence of failures was determined by dynamic observations during the mechanical test.

**Figure 20:**
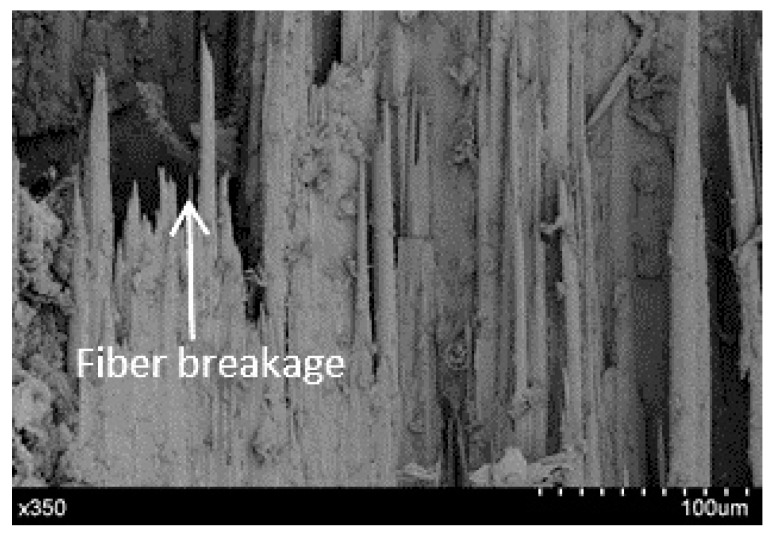
Tensile failure mode.

The SEM photomicrograph of Figure 20 represents the local morphology and structure of the composite, which depicts that the tensile fracture of the specimen under quasi-static loading is due mainly to explosive fiber breakage. The first crack appears in the node region upon a failure in the fiber. Next, a longitudinal crack appears in the PLA matrix and lignin; finally, another transversal crack appears. These analysis is in agreement with those in other studies on bamboo composites [7].

**Figure 21:**
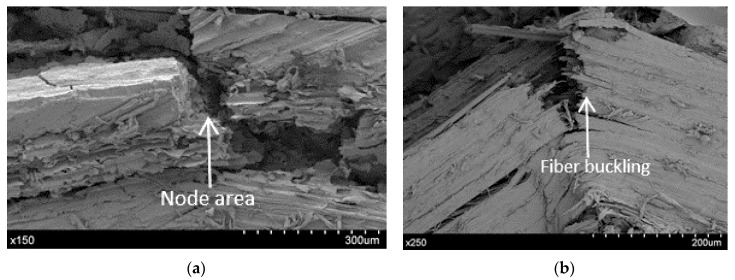
Compressive failure mode, (**a**) Node area failure; (**b**) Fiber bucking failure.

Compressive failure Figure 21 appears in the interface of lignin with the PLA matrix. The bamboo stack breaks at 45° because of the initial buckling stage, as the PLA cannot stabilize all bamboo stacks under compression.

**Figure 22:**
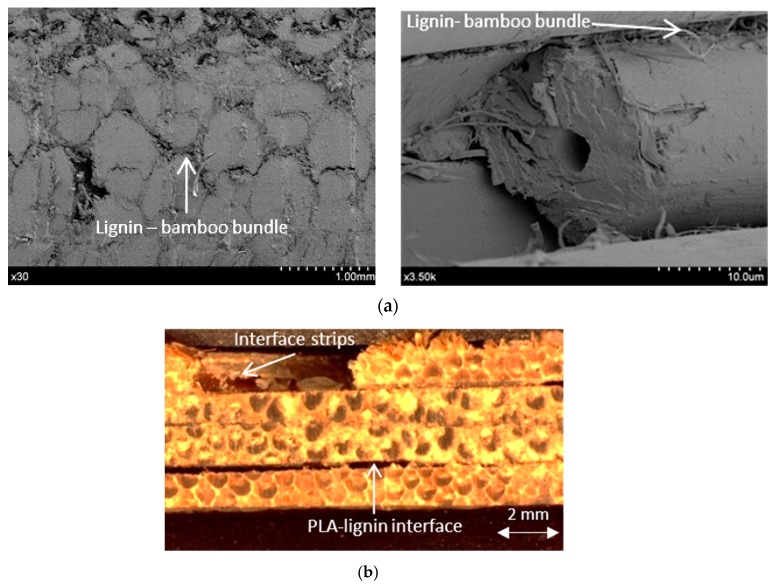
In-plane shear failure mode, (**a**) Lignin- bamboo bundle failure; (**b**) PLA-lignin failure.

For the in-plane shear failure mode Figure 22, the cracks grow through the interfaces between the PLA resin and lignin matrix of the bamboo stacks and the interface between the bamboo fiber bundles and lignin.

**Figure 23:**
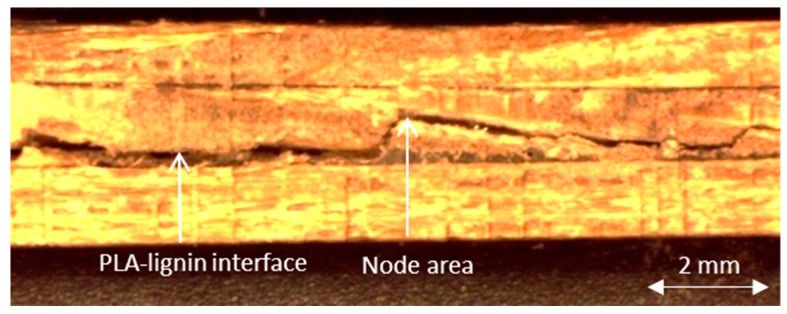
Four-point bending failure mode.

The flexure failure mode Figure 23 appears in the interface between layers, at the edges of the specimens. This failure is not considered permissible by the standards. 

## 5. Conclusions

The main goal of this study was to develop a biodegradable composite for structural applications. This was achieved through the combination of the proper species of bamboo reinforcement and suitable PLA biopolymer, the development of an innovative extraction process, and the optimization of the parameters involved in the manufacturing process.

The critical issue in the manufacturing process of bamboo-based composites is the extraction of the natural fiber. Different processes to extract the fiber can be found in the literature, such as steam explosion and chemical treatments. However, all are high-cost in terms of energy and environmental impact. The innovative mechanical extraction process developed in this study can extract natural strip reinforcements with high performance, low cost, and high rate, with no negative environmental impact, as no chemical treatments are used. The process can be performed at the industrial level.

The mechanical tests performed on the bamboo-based composite showed very good mechanical behavior, better than expected because of the node influence. After this study, the fully biodegradable composite material of bamboo–PLA could be considered a worthy alternative to the E-Glass/Epoxy composite (considered as a reference in this study), wood-reinforced materials, and aluminum alloys. Since the bamboo composite material has a low density of 0.91 g/cm^3^, the specific tensile, compressive, and bending strengths are similar to those of the reference. Moreover, it should be pointed out that the specific Young modulus of the bamboo–PLA composite of 38 GPa·cm^3^/g is more than twice that of the reference at 18 GPa·cm^3^/g.

It was demonstrated that the manufacturing process had a dramatic impact on the mechanical properties. Despite the mechanical properties of strips containing nodes being decreased by approximately 30% relative to those of node-free strips, the optimizations introduced in the manufacturing process compensate for the presence of nodes in the overall properties, while assuring low standard deviations and high average tensile mechanical properties.

The specific in-plane shear strength of the biodegradable composite is much lower than that of the E-Glass/Epoxy. This value is mostly related to the poor interfacial properties between the PLA and the natural fiber, and might be improved with chemical or mechanical treatments, but it may be impossible to obtain values comparable to those of the reference. Therefore, because the mechanical properties mostly related to the fiber showed good behavior, other resins such as epoxy or phenolic resin could be used to obtain better interfaces while maintaining good mechanical properties. It is worthwhile to emphasize that the PLA resin content is only 3.5%. Hence, in the case of using other resins, such as epoxy or phenolic resins, to achieve better performance, the composite material would retain eco-friendliness because the non-biodegradable resin content would be low.

Another important parameter analyzed was the behavior of the composite under environmental conditions. The high temperature and relative humidity considered in the aging cycle did not affect the mechanical properties. It should be noted that the maximum temperature considered for the aging cycle is near the *T*_g_ of the PLA material. Therefore, based on the experimental results, it is necessary to modify the parameters to determine the real allowable conditions for the composite material.

The potential applications of the developed material for aerospace are very restricted, but the high mechanical properties could fulfill the requirements of other industrial sectors, such as energy or automotive. Wind turbine blades are typically made of glass fiber composites, and the material could be also employed in some surfaces for cars. Both sectors select structural materials based mainly on the cost. Housing and watercraft could be also potential markets if the loss of mechanical properties with exposure to humidity could be improved by the addition of a coating, for example.

## Figures and Tables

**Table 1:** Mechanical properties of natural fibers. Values are given for elementary fiber or bundle test, primarily taken from [4,5].

Fiber	Density (g/cm^3^)	Tensile Strength (MPa)	Tensile Modulus (GPa)	Reference
Bamboo	0.59–1.10	140–1200	12–32	[6,7,8]
Sisal	1.00–1.50	80–855	9–38	[9,10,11]
Hemp	1.18–1.60	310–110	3–90	[12]
Flax	1.30–1.58	343–1500	8–160	[4,9,13]
Jute	1.43–1.52	187–800	3–64	[14]
Kenaf	1.20–1.40	180–1191	14–128	[15]

**Table 2:** Natural fiber-reinforced composites.

Composite	Tensile Strength (MPa)	Young Modulus (GPa)	Flexural Strength (MPa)	Company
Flax UD */Polyester	198	20	229	Composites Evolution [19]
Flax Biaxial/Polyester	85	8.7	135	
Flax UD/Epoxy	222	30	271	
Flax Mat/Polyethylene	81	8	130	Flax Composites [20]
Flax/Polypropylene	50	5.4	107	
Flax UD/Epoxy	383	32	330	Bcomp [21]
Flax Plain/Epoxy	115	16.2	130	Groupe Depestele [22]
Flax UD/Epoxy	270	28	226	
Flax UD/Epoxy	365	35	300	Lineo [23]
Flax/Polyester	277	23.4	239.2	[14]
Hemp/Epoxy	195	19.0	161.9	[14]
Hemp/Polyester	171.3	17.0	181.2	[14]
Sisal/Epoxy	132.7	0.67	288.6	[24]
Sisal/Polyester	65.5	1.9	99.5	[25]
Sisal/Polyurethane	118.6	1.81	96.1	[26]
Jute/Polyester	175.1	16.1	148.8	[14]
Jute/Epoxy	185.5	15.0	166.6	[14]
Jute UD/Polyester	307	27	321	Composites Evolution [19]
Jute Fabric/Polyester	59	8.1	87	

* UD: Unidirectional Tape.

**Table 3:** Biodegradable composites based on Polylactic acid (PLA).

Composite	Tensile Strength (MPa)	Young Modulus (GPa)	Flexural Strength (MPa)	Company
Flax 2×2/PLA	110	14	123	Composites Evolution [19]
Flax Plain/PLA	99.5	13.7	125.8	Groupe Depestele [22]
Flax/PLA	44–107.0	6.3–8.0	-	[9,27,28]
Kenaf/PLA	60–223	6.4–23.5	254	[15,29]
Jute/PLA	55.3–100.5	1.7–8.5	75.9–84.5	[9,30]
Sisal/PLA	56.7–188.5	3.8–20	80–100	[9,31,32]
Hemp/PLA	73.0	5.89	102.0	[33]

**Table 4:** Bamboo composites.

Composite	Tensile Strength (MPa)	Young Modulus (GPa)	Flexural Strength (MPa)	Company
Laminated Veneer	68.5	7.8	50.8	Bamboocomposites [43]
Bamboo Laminates	129	10.9	-	
Bamboo Flooring	35	-	-	
Flake board	37.5	3	-	Bambooindustry [44]
Bamboo Lumber/Resorcinol	86	12.1	-	[45]
Bamboo/PLA	29–80.6	0.98–5.9	104–149.3	[46,47,48,49]
Bamboo/Polyester	126	2.5	128	[25]
Bamboo/Epoxy	87–205	3–16	107–140	[17,50,51,52]

**Table 5:** Morphological characteristics [53].

Species	Internodal Length (mm)	Nodal Length (mm)	Thickness of Section (mm)	Culm Diameter (mm)	Thickness of Outer Section (mm)	Area of Bundle (mm^2^)	Distance between Bundles (mm)
Moso	250	18	10	90	2	0.35	0.13
Guadua	330	60	21	120	3	0.24	0.11
